# Development of a multidimensional machine learning framework for predicting post‐stroke cognitive impairment: A prospective cohort study

**DOI:** 10.1002/ctm2.70546

**Published:** 2025-12-08

**Authors:** Aini He, Houlin Lai, Xuefan Yao, Benke Zhao, Wenjing Yan, Wei Sun, Xiao Wu, Kehui Ma, Yuan Wang, Haiqing Song

**Affiliations:** ^1^ Department of Neurology Xuanwu Hospital Capital Medical University Beijing China; ^2^ National Engineering Research Centre for Agri‐Product Quality Traceability Beijing Technology and Business University Beijing China; ^3^ Beijing Stroke Quality Control Center Beijing China

1

Dear Editor,

Post‐stroke cognitive impairment (PSCI) remains a prevalent and debilitating complication that profoundly impacts stroke survivors’ quality of life and long‐term outcomes.^1^ Building upon our previous report that 78.7% of Chinese patients with first‐ever ischemic stroke developed PSCI,^2^ we conducted a prospective cohort study to establish an interpretable, multidimensional prediction framework using multiple machine learning (ML) algorithms.

A total of 518 acute ischemic stroke (AIS) patients were recruited at Xuanwu Hospital between January and December 2022. Following rigorous screening, 437 patients completed a 3‐month cognitive follow‐up using the Telephone Interview for Cognitive Status‐40 (TICS‐40), and 190 (43.5%) were identified as having PSCI (see Figure S1 for details). We collected 89 clinical, neuroimaging, and serological variables (see Table S1 for details). The dataset was split 8:2 into training and test sets. All preprocessing steps, namely outlier removal, imputation, and normalisation, were applied exclusively to the training set. Using 10‐fold cross‐validation combined with Recursive Feature Elimination (RFE), six ML algorithms, including Logistic Regression (LR), Decision Tree (DT), Random Forest (RF), Light Gradient Boosting Machine (LightGBM), eXtreme Gradient Boosting (XGBoost) and Categorical Boosting (CatBoost), were trained and optimised. Model performance was subsequently evaluated on the test set (see Supplementary Information for details).

Through the feature selection process, a set of twenty features was identified across the six models (see Figure 1 and Figure S2 for details). Baseline characteristics were compared between the PSCI and non‐PSCI groups in Table S2. Comparative analysis revealed that the gradient boosting models (LightGBM, XGBoost and CatBoost) consistently demonstrated superior comprehensive predictive performance compared to LR, DT and RF across key metrics, including area under the curve (AUC) (0.73–0.77), precision‐recall balance, and clinical net benefit in decision curve analysis (DCA) (see Table 1 and Figure 2 for details). LightGBM and XGBoost excelled in computational efficiency and scalability, whereas CatBoost offered superior stability on limited and imbalanced data. This functional diversity highlights ensemble methods as a robust and adaptable framework for developing clinically viable PSCI predictors.

**FIGURE 1:**
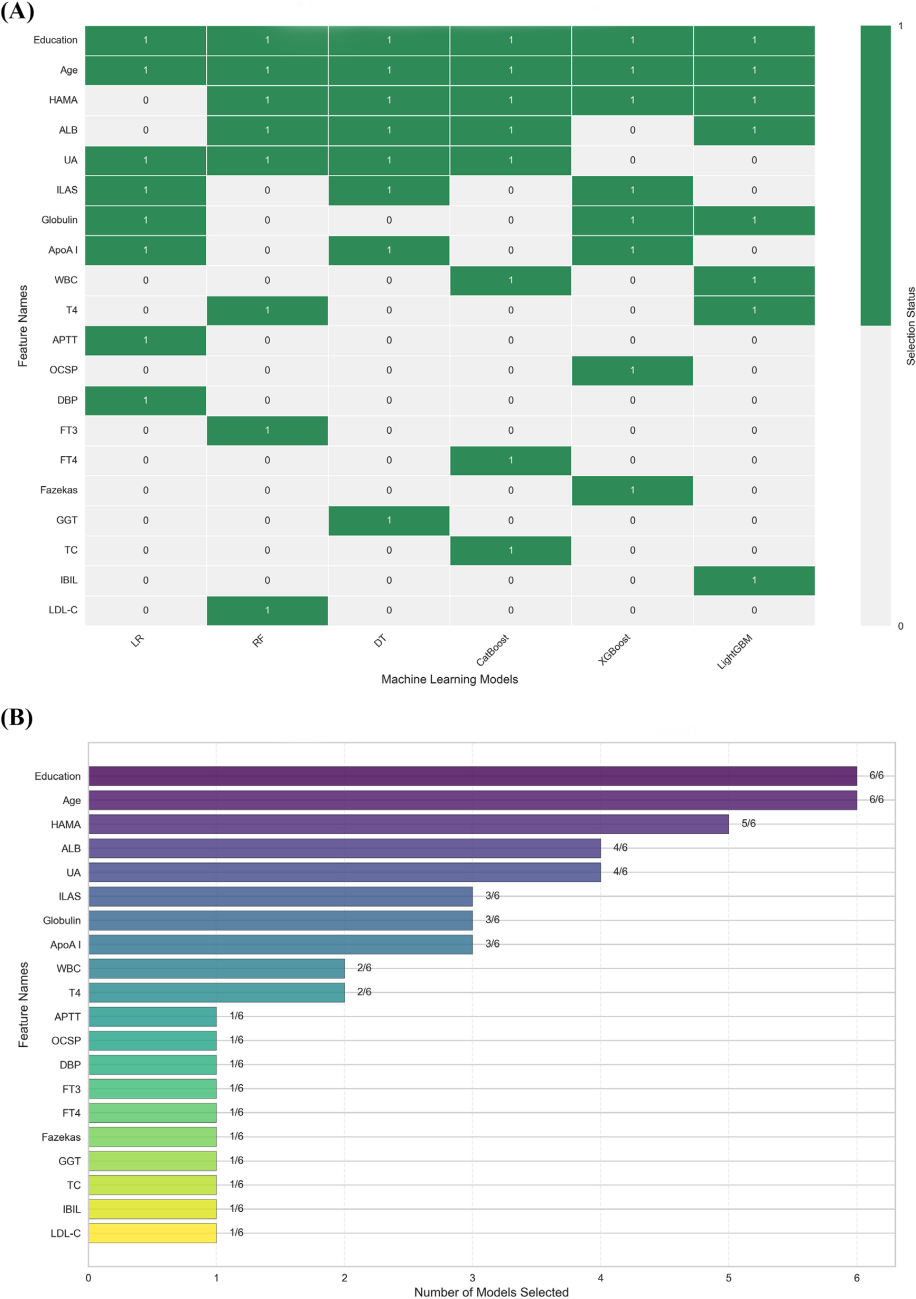
Feature variables selected by the models. (A) Feature selection consistency heatmap across machine learning models. The heatmap illustrates the consistency of selected predictors among six machine learning (ML) algorithms. Each row represents a clinical or biochemical variable, and each column corresponds to a model. A value of 1 (green) indicates that the feature was selected by the respective model, whereas 0 (white) indicates exclusion. The colour bar on the right denotes the binary selection indicator. (B) Feature selection frequency ranking across machine learning models. This bar chart depicts the frequency with which each variable was selected across six machine learning algorithms. The X‐axis indicates the number of models in which each feature was retained, while the Y‐axis lists all candidate variables.

**TABLE 1:** Model performance evaluation using bootstrapping.

Model	AUC	Accuracy	Precision	Recall	F1 score	MCC
LR	0.692 (0.576, 0.794)	0.633 (0.534, 0.727)	0.608 (0.419, 0.786)	0.444 (0.289, 0.595)	0.510 (0.349, 0.647)	0.238 (0.022, 0.432)
DT	0.618 (0.488, 0.738)	0.644 (0.545, 0.750)	0.584 (0.442, 0.737)	0.629 (0.463, 0.790)	0.602 (0.466, 0.723)	0.282 (0.072, 0.496)
RF	0.672 (0.560, 0.776)	0.655 (0.557, 0.761)	0.604 (0.439, 0.762)	0.603 (0.444, 0.757)	0.600 (0.459, 0.725)	0.299 (0.105, 0.510)
LightGBM	0.766 (0.655, 0.856)	0.736 (0.636, 0.818)	0.726 (0.571, 0.875)	0.630 (0.476, 0.765)	0.672 (0.537, 0.784)	0.457 (0.259, 0.629)
Xgboost	0.751 (0.638, 0.848)	0.667 (0.568, 0.761)	0.655 (0.472, 0.828)	0.498 (0.341, 0.649)	0.562 (0.413, 0.696)	0.312 (0.107, 0.510)
CatBoost	0.734 (0.638, 0.848)	0.655 (0.557, 0.750)	0.641 (0.462, 0.818)	0.471 (0.317, 0.629)	0.539 (0.387, 0.677)	0.285 (0.090, 0.488)

Abbreviations: Catboost, Categorical Boosting; DT, Decision Tree; LightGBM, Light Gradient Boosting Machine; LR, Logistic Regression; MCC, Matthews Correlation Coefficient; RF, Random Forest; XGBoost, eXtreme Gradient Boosting.

**FIGURE 2:**
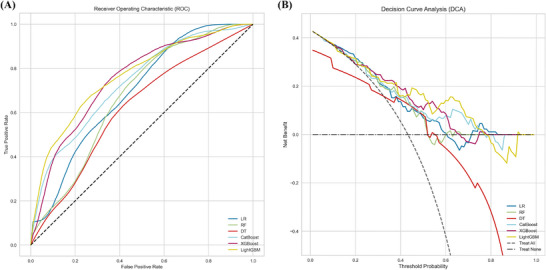
Receiver operating characteristic (ROC) and decision curve analysis (DCA) curves of the models. (A) The ROC curves evaluate the model's performance, with the X‐axis representing the false positive rate and the Y‐axis representing the true positive rate. The diagonal dashed line illustrates the performance of a random classifier with an area under the curve (AUC) of 0.5, serving as a baseline. (B) The DCA curves assess the net benefit of models across different threshold probabilities. The X‐axis representes the decision threshold, while the Y‐axis indicates the net benefit of using the model. The dashed line depictes the net benefit without a model, typically zero or lower, serving as a reference.

All models consistently identified age and education level as core determinants of PSCI (see Figure 1 for details). Ageing is a primary non‐modifiable risk factor that promotes the accumulation of neuropathological proteins, neuroinflammation, lipid dysregulation, and neurodegeneration, culminating in cognitive decline.^3^ In contrast, higher education confers protection, plausibly via mechanisms of cognitive reserve and socioeconomic advantage, sustaining compensatory neural efficiency and resilience to vascular and degenerative insults.^4^

Additionally, psychological and metabolic features were frequently selected across models, including Hamilton Anxiety Scale (HAMA) score, uric acid (UA), and albumin (ALB) (see Figure 1 for details). Evidence suggests that chronic anxiety detrimentally impacts cognition and quality of life in older adults, likely via mitochondrial dysfunction and damage to neuronal axons and synapses.^5^ UA, a major endogenous scavenger of reactive oxygen species, may protect against ischemia‐related oxidative injury and support endothelial function within neurovascular networks.^6^ Pharmacological interventions aimed at lowering UA, such as allopurinol and uricosuric agents, have been studied in relation to dementia; for instance, allopurinol has been explored for vascular dementia and uricosuric agents for Alzheimer's disease. However, these associations diminished after adjusting for established dementia risk factors.^7^ Biochemically, ALB exerts pleiotropic neuroprotective actions: by binding and facilitating the clearance of β‐amyloid species, it may mitigate amyloid aggregation and downstream neurotoxicity. Concurrently, the antioxidant, anti‐inflammatory, and anti‐tau properties of ALB contribute to the preservation of synaptic integrity and the inhibition of neurodegenerative pathways.^8^, ^9^

Several additional variables—including intracranial large artery stenosis, globulin, apolipoprotein A‐I, white blood cell count, thyroxine (T4), free thyroxine (FT4), free triiodothyronine (FT3), diastolic blood pressure, activated partial thromboplastin time, gamma‐glutamyl transferase, low‐density lipoprotein cholesterol, indirect bilirubin, Oxford Cognitive Screen Plus, Fazekas grade and total cholesterol—were identified in individual models only (see Figure 1 for details). Their inconsistent selection across models could stem from complex interdependencies and higher‐order interactions, suggesting they operate within a network of risk factors rather than in isolation (see Figure S3 for details). These factors may therefore reflect latent predictive signals and reveal novel, multifactorial mechanisms in PSCI, meriting further investigation.

In conclusion, ML frameworks consistently reveal a core set of demographic, psychological, vascular, and metabolic determinants underlying PSCI, emphasising its multifactorial, network‐level vulnerability. By integrating complex biological and clinical data, these approaches enhance clinical interpretability, guide precision prevention and inform individualised, multimodal interventions targeting the vascular‐metabolic‐neural axis. Collectively, these findings deepen our understanding of PSCI pathophysiology and provide a roadmap for mechanism‐informed therapeutic strategies and future translational research.

## AUTHOR CONTRIBUTIONS

Aini He developed the model and wrote the first draft of the manuscript. Haiqing Song and Yuan Wang contributed to the study concept and design, had full access to all study data, and were responsible for the integrity of the data and the accuracy of the analysis. Houlin Lai, Xuefan Yao, Xiao Wu, and Kehui Ma screened patients and collected baseline data, while Benke Zhao and Wei Sun acquired follow‐up information. Aini He, Houlin Lai, and Wenjing Yan performed the statistical analysis. All authors provided administrative, technical, or material support, and approved the final manuscript.

## CONFLICT OF INTEREST STATEMENT

The authors declare no conflict of interest.

## FUNDING INFORMATION

This study was supported by the National Key Research and Development Program of China (2022YFC3600504), the Science and Technology Innovation 2030‐Major Project (2021ZD0201806), the Beijing Nova Program (20240484612) and the Key Program of the National Natural Science Foundation of China (62433002).

## ETHICS STATEMENT

The study was approved by the Ethics Review Board of Xuanwu Hospital, Capital Medical University (No. 临研审[2021]053).

## INFORMED CONSENT STATEMENT

Written informed consent was obtained from all subjects before the study.

## Supporting information



Supporting Information

## Data Availability

Detailed data are available from the corresponding author on reasonable request.
