# Supplementary material for: Development of a multidimensional machine learning framework for predicting post‐stroke cognitive impairment: A prospective cohort study
Source: Clin Transl Med. 2025 Dec 8;15(12):e70546. doi: 10.1002/ctm2.70546 (PMC12685603; doi:10.1002/ctm2.70546)
Supplement: Supplementary file 1 — Supporting Information [file CTM2-15-e70546-s001.docx]

**Supplementary Information**

Methods

Study design and participants

This prospective cohort study recruited acute ischemic stroke (AIS) patients from Xuanwu Hospital, Capital Medical University, between January and December 2022. AIS patients were identified and diagnosed according to the American Heart Association/American Stroke Association definition.^1^ The adherence to the principles outlined in the Declaration of Helsinki was maintained. Informed consent was signed by all participants and approved by the Research Ethics Committee.

The inclusion criteria were as follows: (1) patients aged ≥ 18 years, (2) a diagnosis of AIS confirmed by DWI, (3) onset of symptom within 7 days, and (4) willingness to participate in the cognitive function assessments and adhere to the scheduled follow-up plan. The exclusion criteria included: (1) silent infarction or transient ischemic attack, (2) hemorrhage or subarachnoid hemorrhage, (3) recurrent stroke during the follow-up period, (4) pre-existing cognitive impairment and other neurological disorders (e.g., depression, Parkinson's disease), (5) severe systemic conditions with a life expectancy of less than two years, and (6) conditions interfering with assessments, such as severe aphasia (defined as a score ≥ 2 on the "aphasia" sub-test of the National Institutes of Health Stroke Scale [NIHSS]), severe hearing impairment, significant disturbances in consciousness, or other similar conditions.

**Baseline data collection**

We collected demographic information, vascular risk factors, admission status, stroke characteristics, and serological biomarkers. Demographics included age, gender, education level, occupation, marital status, living alone, handedness (dextral), and place of residence. Vascular risk factors comprised histories of hypertension, diabetes, hyperlipidemia, coronary heart disease (CHD), atrial fibrillation (AF), long-term smoking, alcoholism, and previous stroke, as well as the use of antihypertensive drugs, hypoglycemic agents, lipid-lowering drugs, and antithrombotic drugs. Information on admission status and stroke characteristics was also collected, including body mass index (BMI), systolic blood pressure (SBP), diastolic blood pressure (DBP), heart rate (HR), National Institutes of Health Stroke Scale (NIHSS) score, modified Rankin Scale (mRS) score, Trial of Org 10172 in Acute Stroke Treatment (TOAST) classification, and Oxfordshire Community Stroke Project (OCSP) classification. Acute reperfusion therapies (intravenous thrombolysis, endovascular intervention) and in-hospital complications (hemorrhage, pneumonia) were documented. Regarding imaging, we primarily gathered lesion volume,^2^ Fazekas grade, the number of chronic lacunes, the degree of intracranial large artery stenosis (ILAS), the presence of the strategic infarction,^3^ dominant hemisphere, and brain atrophy. Serological biomarkers were included according to the previous literature, as detailed in Supplementary Table 1.

*Neuropsychological assessment*

On day 90 after the stroke event (with a standard deviation allowance of +7 days), patients underwent a neuropsychological assessment. This study used the Telephone Interview of Cognitive Status-40 (TICS-40) to assess cognitive function in ischemic stroke patients. Based on established standards in the literature, a TICS-40 score was set at 20 as the cutoff for potential cognitive impairment. Patients scores of 20 and below were categorized as possibly having post-stroke cognitive impairment(PSCI), while those scores above 20 were categorized as the Non-PSCI group.^4^ Simultaneously, the Pittsburgh Sleep Quality Index (PSQI), Hamilton Anxiety Scale (HAMA), and Hamilton Depression Scale (HAMD) were administered.

Statistical analysis

All data from this study were analyzed using python version 3.9 (Python Software Foundation). Continuous variables were reported as mean ± standard deviation or as medians with interquartile ranges, depending on the distribution. Categorical variables were presented as counts and percentages. Comparisons between groups were conducted using t-tests or Mann–Whitney U tests for continuous variables, and chi-squared tests or Fisher’s exact tests for categorical variables, as appropriate.

Data preprocessing

The collected follow-up data were imported into the Python environment and randomly divided into training and test sets in an 8:2 ratio for model development and evaluation.

For the training set, missing data were handled through imputation based on variable types. Continuous variables (e.g., age, NIHSS, PSQI, HAMD, and HAMA) were imputed with mean values, while ordered discrete variables, such as education level, Fazekas grade, and ILAS, were imputed with median values. Unordered categorical variables, including gender, occupation, marital status, living alone status, handedness, residence, and vascular risk factors, were imputed using the mode. Continuous variables were standardized using the Z-score method to ensure scale consistency.

Model building

With cognitive impairment as the dependent variable, a total of 89 clinical and imaging variables were included in the development of the ML models. Logistic Regression (LR) was utilized for binary classification, providing interpretable feature coefficients to reveal variable relationships. Decision Trees (DT) provided interpretability and mixed-data versatility but were prone to overfitting, especially with limited data. Random Forest (RF) reduced overfitting through ensemble averaging of multiple trees, improving generalization. Light Gradient Boosting Machine (LightGBM) prioritized speed and efficiency for large-scale data with missing values, while XGBoost emphasized regularization and scalability of tabular tasks. Categorical Boosting (CatBoost) specialized in automatic processing of categorical features, minimizing preprocessing requirements.^5-7^

To ensure optimal predictive performance and model interpretability, Recursive Feature Elimination (RFE) was applied using model-derived importance measures to systematically identify the most informative features. Features were ranked by importance, and the top 10% were retained to construct a reduced feature set. A second round of training was then conducted using this refined feature subset, with ten-fold cross-validation employed for hyperparameter tuning.

Model evaluation

Based on the confusion matrix, metrics such as accuracy, precision, recall, F1 score and Matthews Correlation Coefficient (MCC) were calculated to assess the effectiveness of the ML models. The model's ability to classify was assessed by the Receiver Operating Characteristic (ROC) curve, which plotted the true positive rate versus false positive rate across various thresholds to provide a visual overview of classification performance. The Area Under the Curve (AUC) quantified the overall discriminative ability of the model. Additionally, clinical utility was analyzed via Decision Curve Analysis (DCA), comparing net benefit against threshold probabilities.

1. Powers WJ, Rabinstein AA, Ackerson T*, et al*. Guidelines for the Early Management of Patients With Acute Ischemic Stroke: 2019 Update to the 2018 Guidelines for the Early Management of Acute Ischemic Stroke: A Guideline for Healthcare Professionals From the American Heart Association/American Stroke Association. *Stroke*. Dec 2019;50(12):e344-e418. doi:10.1161/STR.0000000000000211

2. Kissela B BJ, Woo D, Kothari R, Miller R, Khoury J, Brott T, Pancioli A, Jauch E, Gebel J, Shukla R, Alwell K, Tomsick T. Greater Cincinnati/Northern Kentucky Stroke Study: volume of first-ever ischemic stroke among blacks in a population-based study. *Stroke*. 2001:Jun;32(6):1285-90. doi:10.1161/01.str.32.6.1285

3. Weaver NA, Kuijf HJ, Aben HP*, et al*. Strategic infarct locations for post-stroke cognitive impairment: a pooled analysis of individual patient data from 12 acute ischaemic stroke cohorts. *Lancet Neurol*. Jun 2021;20(6):448-459. doi:10.1016/S1474-4422(21)00060-0

4. Liu Y-H, Chen Y, Wang Q-H*, et al*. One-Year Trajectory of Cognitive Changes in Older Survivors of COVID-19 in Wuhan, China. *JAMA Neurology*. 2022;79(5)doi:10.1001/jamaneurol.2022.0461

5. Chen IY, Joshi S, Ghassemi M, Ranganath R. Probabilistic Machine Learning for Healthcare. *Annu Rev Biomed Data Sci*. Jul 20 2021;4:393-415. doi:10.1146/annurev-biodatasci-092820-033938

6. Ahn JM, Kim J, Kim K. Ensemble Machine Learning of Gradient Boosting (XGBoost, LightGBM, CatBoost) and Attention-Based CNN-LSTM for Harmful Algal Blooms Forecasting. *Toxins (Basel)*. Oct 10 2023;15(10)doi:10.3390/toxins15100608

7. Belsti Y, Moran L, Du L*, et al*. Comparison of machine learning and conventional logistic regression-based prediction models for gestational diabetes in an ethnically diverse population; the Monash GDM Machine learning model. *Int J Med Inform*. Nov 2023;179:105228. doi:10.1016/j.ijmedinf.2023.105228

**Supplementary Table 1. Abbreviation of characteristic variables**

|  | **Characteristic variables** |
| --- | --- |
| Vascular risk factors | Coronary heart disease (CHD), Atrial fibrillation (AF). |
| Admission status | Body Mass Index (BMI), Systolic pressure (SBP), Diastolic pressure (DBP), Heart rate (HR). |
| Stroke characteristics | National Institute of Health stroke scale (NIHSS), Modified Rankin Scale (mRS), Trial of Org 10172 in Acute Stroke Treatment (TOAST), Large artery atherosclerosis (LAA), Small artery occlusion (SAO), Cardioembolism (CE), Oxfordshire community stroke project (OCSP), Total anterior circulation infarcts (TACI), Partial anterior circulation infarcts (PACI), Posterior circulation infarcts (POCI), Lacunar infarcts (LACI), Intracranial large artery stenosis (ILAS). |
| Serological biomarkers | Glycosylated Hemoglobin (HbA1c), Fasting Blood Glucose (FBG), Total Protein (TP), Albumin (ALB), Globulin (GLOB), Total Cholesterol (TC), Triglycerides (TG), High-Density Lipoprotein Cholesterol (HDL-C), Low-Density Lipoprotein Cholesterol (LDL-C), Apolipoprotein AⅠ(ApoAⅠ), Apolipoprotein B (ApoB), Total Bilirubin (TBIL), Direct Bilirubin (DBIL), Indirect Bilirubin (IBIL), Total Bile Acids (TBA), Gamma-Glutamyl Transferase (GGT), Alkaline Phosphatase (ALP), Alanine Aminotransferase (ALT), Aspartate Aminotransferase (AST), Lactate Dehydrogenase (LDH), Alpha-Hydroxybutyrate Dehydrogenase (α-HBDH), Creatine Kinase (CK), Urea, Creatinine (Cr), Uric Acid (UA), High-Sensitivity C-Reactive Protein (hs-CRP), Homocysteine (Hcy), Hemoglobin (Hb), Red Blood Cell Count (RBC), White Blood Cell Count (WBC), Lymphocyte Count (LYM), Platelet Count (PLT), International Normalized Ratio (INR), Thrombin Time (TT), Prothrombin Time Activity (PTA), Activated Partial Thromboplastin Time (APTT), Prothrombin Time (PT), Fibrinogen (FIB), D-dimer, Antithrombin III (AT III), Free Triiodothyronine (FT3), Thyroid-Stimulating Hormone (TSH), Triiodothyronine (T3), Thyroxine (T4), Free Thyroxine (FT4), Erythrocyte Sedimentation Rate (ESR), C-Reactive Protein (CRP). |
| Neuropsychological assessment | Telephone Interview of Cognitive Status-40 (TICS-40), Hamilton Anxiety Scale (HAMA), Hamilton Depression Scale (HAMD), Pittsburgh sleep quality index (PSQI). |

**Supplementary Table 2. Cohort characteristics**

|  | **Total** | **PSCI** | **Non-PSCI** | **P value** |
| --- | --- | --- | --- | --- |
|  |  |  |  |  |
| **Number (%)** | 437 | 190 (43%) | 247 (57%) |  |
| **Demographic information** | | | | |
| Age (yrs) | 63.0 (54.0, 69.0) | 66.0 (59.0, 70.8) | 60.0 (50.0, 67.0) | ＜0.001* |
| Male (%) | 323 (74%) | 134 (71%) | 189 (77%) | 0.192 |
| Dextral (%) | 404 (92%) | 178 (94%) | 226 (91%) | 0.500 |
| Living alone (%) | 45 (10%) | 21 (11%) | 24 (10%) | 0.767 |
| Marital status (%) |  |  |  | 1.000 |
| Married | 376 (86%) | 163 (86%) | 213 (86%) |  |
| Unmarried, divorced or widowed | 61 (14%) | 27 (14%) | 34 (14%) |  |
| Residence (%) | | | | 0.005* |
| Urban | 354 (81%) | 142 (75%) | 212 (86%) |  |
| Rural | 83 (19%) | 48 (25%) | 35 (14%) |  |
| Pre-retirement occupation (%) | | | | 0.102 |
| Mental worker | 273 (62%) | 110 (58%) | 163 (66%) |  |
| Labor worker | 164 (38%) | 80 (42%) | 84 (34%) |  |
| Education (%) |  |  |  | ＜0.001* |
| 0-6 yrs | 51 (12%) | 39 (21%) | 12 (5%) |  |
| 7-9 yrs | 151 (35%) | 74 (39%) | 77 (31%) |  |
| 10-12 yrs | 142 (32%) | 57 (30%) | 85 (34%) |  |
| ≥13 yrs | 93 (21%) | 20 (11%) | 73 (30%) |  |
| **Vascular risk factors** | | | | |
| Hypertension (%) | 316 (72%) | 138 (73%) | 178 (72%) | 0.981 |
| Diabetes (%) | 182 (42%) | 86 (45%) | 96 (39%) | 0.212 |
| Hyperlipidemia (%) | 278 (64%) | 120 (63%) | 158 (64%) | 0.519 |
| CHD (%) | 108 (25%) | 59 (31%) | 49 (20%) | 0.013* |
| AF (%) | 39 (9%) | 25 (13%) | 14 (6%) | 0.012* |
| Antihypertensive drugs (%) | 223 (51%) | 100 (53%) | 123 (50%) | 0.623 |
| Hypoglycemic agent (%) | 143 (33%) | 69 (36%) | 74 (30%) | 0.193 |
| Lipid-lowering drugs (%) | 117 (27%) | 50 (26%) | 67 (27%) | 0.936 |
| Antithrombotic drugs (%) | 87 (20%) | 41 (22%) | 46 (19%) | 0.518 |
| Smoking (%) | 245 (56%) | 107 (56%) | 138 (56%) | 1.000 |
| Alcoholism (%) | 163 (37%) | 68 (36%) | 95 (38%) | 0.636 |
| Previous stroke (%) | 130 (30%) | 63 (33%) | 67 (27%) | 0.207 |
| **Admission status** | | | | |
| BMI (kg/m2) | 25.1 (23.0, 27.7) | 24.8 (22.9, 27.3) | 25.4 (23.1, 27.7) | 0.182 |
| SBP (mmHg) | 148.0 (134.0, 162.0) | 148.0 (136.0, 164.0) | 148.0 (134.0, 162.0) | 0.527 |
| DBP (mmHg) | 85.6±12.9 | 83.6±12.7 | 87.2±12.9 | 0.004* |
| HR (bpm) | 74.0 (68.0, 82.0) | 73.0 (68.0, 82.0) | 75.0 (68.0, 82.0) | 0.689 |
| **Stroke characteristics** | | | | |
| Hospitalization period (Day) | 7.0 (6.0, 8.0) | 7.0 (6.0, 8.0) | 7.0 (6.0, 8.0) | 0.075 |
| NIHSS score | 3.0 (1.0, 6.0) | 4.0 (2.0, 7.0) | 3.0 (1.0, 5.0) | 0.002* |
| mRS score | 2.0 (1.0, 4.0) | 2.0 (1.0, 4.0) | 1.0 (1.0, 3.0) | 0.001* |
| Number of lacunes | 0.0 (0.0, 1.0) | 0.0 (0.0, 1.0) | 0.0 (0.0, 0.0) | 0.039* |
| Lesion volume (mm3) | 195.0 (46.0, 1301.0) | 262.0 (54.0, 1882.0) | 150.0 (41.0, 819.0) | 0.045* |
| Revascularization (%) | 168 (38%) | 73 (38%) | 95 (38%) | 1.000 |
| Strategic infarct^†^ (%) | 131 (30%) | 71 (37%) | 60 (24%) | 0.004* |
| Dominant hemisphere (%) | 205 (47%) | 100 (53%) | 105 (43%) | 0.045* |
| Atrophy (%) | 140 (32%) | 82 (43%) | 58 (23%) | ＜0.001* |
| Hemorrhage (%) | 34 (8%) | 19 (10%) | 15 (6%) | 0.181 |
| Pneumonia (%) | 109 (25%) | 65 (34%) | 44 (18%) | ＜0.001* |
| TOAST (%) |  |  |  | ＜0.001* |
| LAA | 285 (65%) | 145 (76%) | 140 (57%) |  |
| SAO | 85 (19%) | 25 (13%) | 60 (24%) |  |
| CE | 35 (8%) | 15 (8%) | 20 (8%) |  |
| Other | 32 (7%) | 5 (3%) | 27 (11%) |  |
| OCSP (%) |  |  |  | 0.006* |
| TACI | 26 (6%) | 14 (7%) | 12 (5%) |  |
| PACI | 123 (28%) | 67 (35%) | 56 (23%) |  |
| LACI | 146 (33%) | 50 (26%) | 96 (39%) |  |
| POCI | 142 (32%) | 59 (31%) | 83 (34%) |  |
| Fazekas (%) |  |  |  | ＜0.001* |
| 0 grade | 36 (8%) | 9 (5%) | 27 (11%) |  |
| 1 grade | 194 (44%) | 74 (39%) | 120 (49%) |  |
| 2 grade | 129 (30%) | 57 (30%) | 72 (29%) |  |
| 3 grade | 78 (18%) | 50 (26%) | 28 (11%) |  |
| ILAS (%) |  |  |  | ＜0.001* |
| <50% | 134 (31%) | 37 (19%) | 97 (39%) |  |
| 50%~69% | 47 (11%) | 21 (11%) | 26 (11%) |  |
| 70%~99% | 130 (30%) | 61 (32%) | 69 (28%) |  |
| 100% | 126 (29%) | 71 (37%) | 55 (22%) |  |
| **Serological biomarkers** | | | | |
| ALB (g/L) | 38.60 (36.40, 40.60) | 37.90 (35.50, 39.70) | 39.30 (37.00, 41.10) | ＜0.001* |
| GLOB (g/L) | 26.50 (24.10, 29.10) | 27.20 (24.60, 29.90) | 26.00 (23.70, 28.50) | 0.005* |
| TG (mmol/L) | 1.39 (0.99, 1.93) | 1.29 (0.92, 1.83) | 1.49 (1.06, 2.06) | 0.016* |
| UA (umol/L) | 313.00 (263.00, 376.00) | 298.00 (249.00, 343.00) | 328.00 (278.00, 398.00) | ＜0.001* |
| Hb (g/L) | 139.00 (128.00, 149.00) | 137.00 (124.00, 147.00) | 140.00 (131.00, 150.00) | 0.006* |
| RBC (*1012/L) | 4.47 (4.09, 4.82) | 4.39 (4.00, 4.74) | 4.52 (4.22, 4.90) | 0.002* |
| INR (INR) | 0.99 (0.95, 1.04) | 1.00 (0.95, 1.06) | 0.99 (0.94, 1.02) | 0.014* |
| PTA (%) | 102.00 (94.00, 110.00) | 100.00 (91.00, 109.00) | 103.00 (96.00, 111.00) | 0.016* |
| PT (s) | 13.20 (12.70, 13.70) | 13.30 (12.80, 13.80) | 13.10 (12.70, 13.60) | 0.011* |
| D-dimer (ug/ml) | 0.40 (0.20, 1.10) | 0.50 (0.30, 1.50) | 0.30 (0.20, 1.00) | 0.002* |
| AT III (%) | 99.00 (88.00, 108.00) | 96 (86.00, 106.00) | 100.00(91.00, 109.00) | 0.010* |
| FT3 (pg/ml) | 3.01 (2.71, 3.31) | 2.91 (2.65, 3.27) | 3.05 (2.80, 3.34) | 0.001* |
| ESR (mm/h) | 7.00 (4.00, 13.00) | 8.00 (5.00, 16.80) | 6.00 (3.00, 12.00) | 0.001* |
| CRP (mg/L) | 3.00 (2.00, 7.00) | 3.00 (2.00, 8.00) | 3.00 (2.00,6.00) | 0.036* |
| Hs-CRP (mg/L) | 2.00 (1.00, 6.00) | 2.00 (1.00, 8.00) | 2.00 (1.00, 5.00) | 0.018* |
| HbA1c (%) | 5.90 (5.50, 7.10) | 6.00 (5.50, 7.10) | 5.80 (5.40, 7.00) | 0.093 |
| FBG (mmol/L) | 5.30 (4.60, 6.90) | 5.40 (4.70, 7.30) | 5.20 (4.60, 6.60) | 0.134 |
| TP (g/L) | 65.00 (61.00, 68.00) | 65.00 (61.00, 68.00) | 65.4 (61.50, 68.40) | 0.403 |
| TC (mmol/L) | 3.95 (3.38, 4.68) | 3.92 (3.19, 4.56) | 3.97 (3.43, 4.75) | 0.173 |
| HDL-C (mmol/L) | 1.05 (0.89, 1.22) | 1.06 (0.90, 1.24) | 1.04 (0.88, 1.21) | 0.485 |
| LDL-C (mmol/L) | 2.30 (1.77, 2.99) | 2.23 (1.73, 2.87) | 2.33 (1.81, 3.00) | 0.139 |
| ApoAⅠ(g/L) | 1.17 (1.06, 1.30) | 1.17 (1.06, 1.30) | 1.19 (1.07, 1.30) | 0.393 |
| ApoB (g/L) | 0.83 (0.70, 1.00) | 0.82 (0.67, 0.96) | 0.84 (0.71, 1.01) | 0.103 |
| TBIL (umol/L) | 13.6 (10.2, 17.2) | 13.6 (10.0, 17.6) | 13.6 (10.6, 16.9) | 0.672 |
| DBIL (umol/L) | 4.40 (3.20, 5.80) | 4.50 (3.20, 6.40) | 4.30 (3.20, 5.50) | 0.371 |
| IBIL (umol/L) | 9.10 (7.00, 11.70) | 9.00 (6.50, 11.60) | 9.20 (7.20, 11.70) | 0.229 |
| TBA (umol/L) | 3.70 (2.20, 5.60) | 4.00 (2.70, 5.70) | 3.30 (2.10, 5.40) | 0.057 |
| GGT (IU/L) | 25.00 (18.00, 37.00) | 24.00 (17.00, 35.00) | 26.00 (19.00, 38.00) | 0.109 |
| ALP (IU/L) | 68.00 (56.00, 81.00) | 66.00 (56.00, 82.00) | 69.00 (57.00, 80.00) | 0.764 |
| ALT (IU/L) | 17.00 (13.00, 25.00) | 16.00 (12.00, 22.00) | 17.00 (13.00, 26.00) | 0.197 |
| AST (IU/L) | 17.00 (14.00, 21.00) | 17.00 (14.00, 22.00) | 17.00 (15.00, 21.00) | 0.834 |
| LDH (IU/L) | 161.00 (142.00, 187.00) | 162.00 (144.00, 191.00) | 159.00 (141.00, 181.00) | 0.156 |
| α-HBDH (IU/L) | 117.00 (101.00, 137.00) | 119.00 (103.00, 141.00) | 116.00 (100.00, 134.00) | 0.200 |
| CK (IU/L) | 64.00 (47.00, 92.00) | 62.00 (40.00, 86.00) | 65.00 (49.00, 98.00) | 0.191 |
| Urea (mmol/L) | 5.00 (4.00, 6.00) | 5.00 (4.00, 6.00) | 5.00 (4.00, 6.00) | 0.262 |
| Cr (umol/L) | 60.00 (50.00, 68.00) | 59.00 (50.00, 67.00) | 60.00 (52.00, 70.00) | 0.554 |
| Hcy (umol/L) | 12.40 (10.10, 16.50) | 12.30 (10.30, 15.90) | 12.50 (10.10, 16.90) | 0.417 |
| WBC (*10^9^/L) | 7.00 (5.70, 8.20) | 7.00 (5.80, 8.40) | 7.00 (5.70, 8.00) | 0.273 |
| LYM (*10^9^/L) | 1.62 (1.25, 2.08) | 1.60 (1.25, 2.08) | 1.64 (1.25, 2.08) | 0.465 |
| Plt (*10^9^/L) | 207.00 (172.00, 255.00) | 206.00 (169.00, 246.00) | 208.00 (176.00, 262.00) | 0.083 |
| TT (s) | 17.00 (16.00, 18.00) | 17.10 (16.30, 18.10) | 17.00 (16.00, 18.00) | 0.477 |
| APTT (s) | 35.00 (33.00, 39) | 35.20 (32.30, 38.90) | 35.00 (33.00, 38.00) | 0.691 |
| FIB (g/L) | 3.06 (2.57, 3.68) | 3.08 (2.57, 3.77) | 3.06 (2.58, 3.65) | 0.595 |
| TSH (uIU/ml) | 1.50 (0.90, 2.50) | 1.50 (0.90, 2.50) | 1.50 (0.90, 2.50) | 0.858 |
| T3 (ng/ml) | 1.01 (0.83, 2.40) | 0.99 (0.83, 2.42) | 1.02 (0.83, 2.38) | 0.492 |
| T4 (ug/dl) | 7.70 (6.40, 9.00) | 7.90 (6.50, 9.00) | 7.60 (6.40, 9.10) | 0.812 |
| FT4 (ng/dl) | 1.11 (1.01, 1.24) | 1.10 (1.00, 1.22) | 1.11 (1.02, 1.25) | 0.309 |
| **Neuropsychological assessment** | | | | |
| HAMA score | 3.8 (2.0, 5.0) | 4.0 (3.0, 5.0) | 3,0 (2.0, 4.0) | ＜0.001* |
| HAMD score | 4.0 (2.0, 5.0) | 4.0 (3.0, 5.0) | 4.0 (2.0, 6.0) | 0.631 |
| PSQI score | 4.0 (3.0, 6.0) | 4.0 (3.0, 6.0) | 4.0 (3.0, 6.0) | 0.085 |
| TICS-40 score | 22.0 (18.0, 25.0) | 18.0 (15.0, 19.0) | 24.0 (22.0, 27.0) | ＜0.001* |

*: Indicated statistical significance at P < 0.05. †: Patients with strategic infarct, defined as infarction involving the left frontal and temporal lobes, left thalamus, and right parietal lobe. PSCI, Post-Stroke Cognitive Impairment. Abbreviations were defined in Supplementary Table 1.

**Supplementary Figure 1. Flowchart of Study Cohort Forming**

**
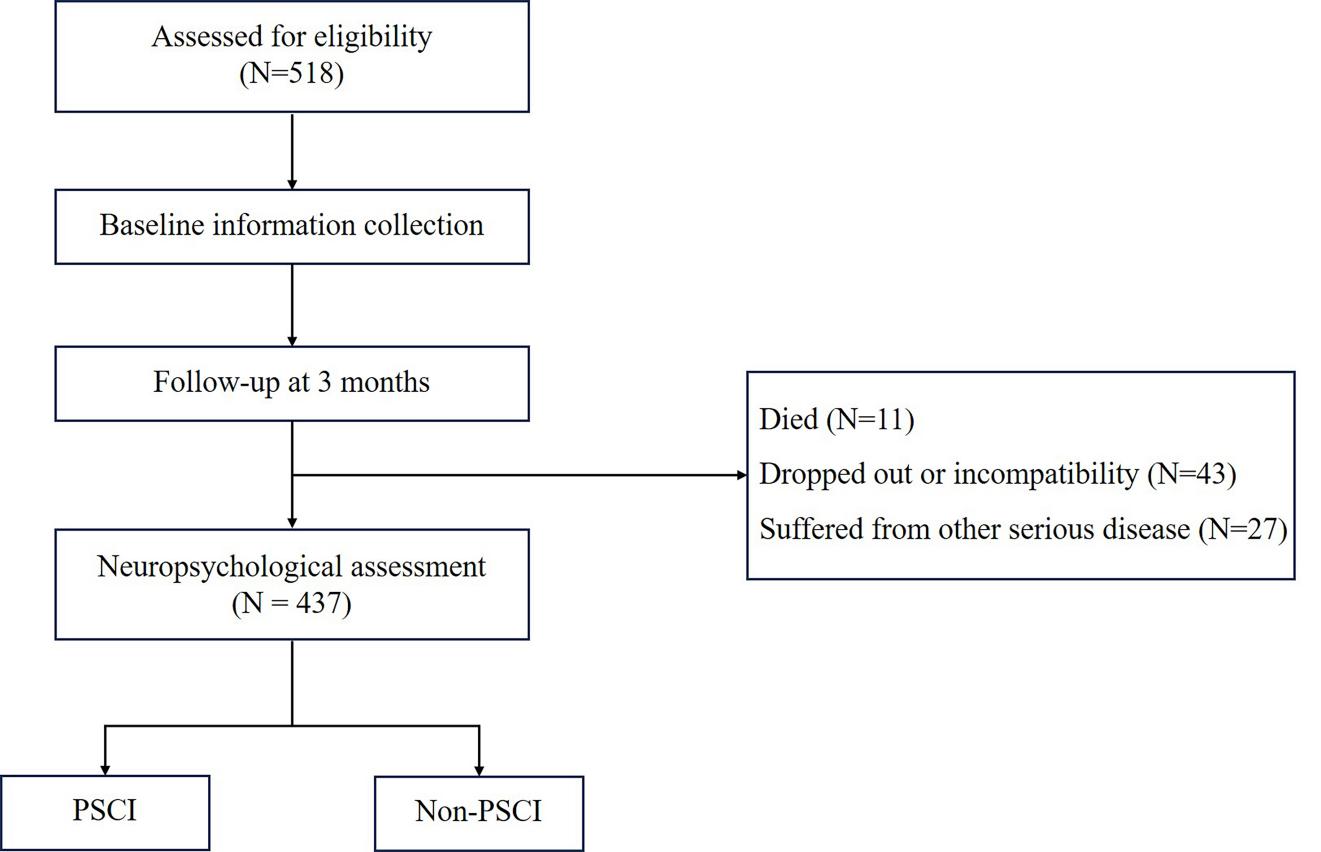
**

Among the 518 patients initially screened for eligibility, baseline demographic and clinical data were collected at enrollment. During the 3-month follow-up period, 81 participants were lost to follow-up: 11 deceased, 43 withdrew or were incompatible with the assessments, and 27 developed other serious medical conditions. Consequently, 437 participants constituted the final cohort for neuropsychological evaluation. These participants were subsequently categorized into post-stroke cognitive impairment (PSCI) and non-PSCI groups for further analyses.

**Supplementary Figure 2. Model Feature Importance Comparison**


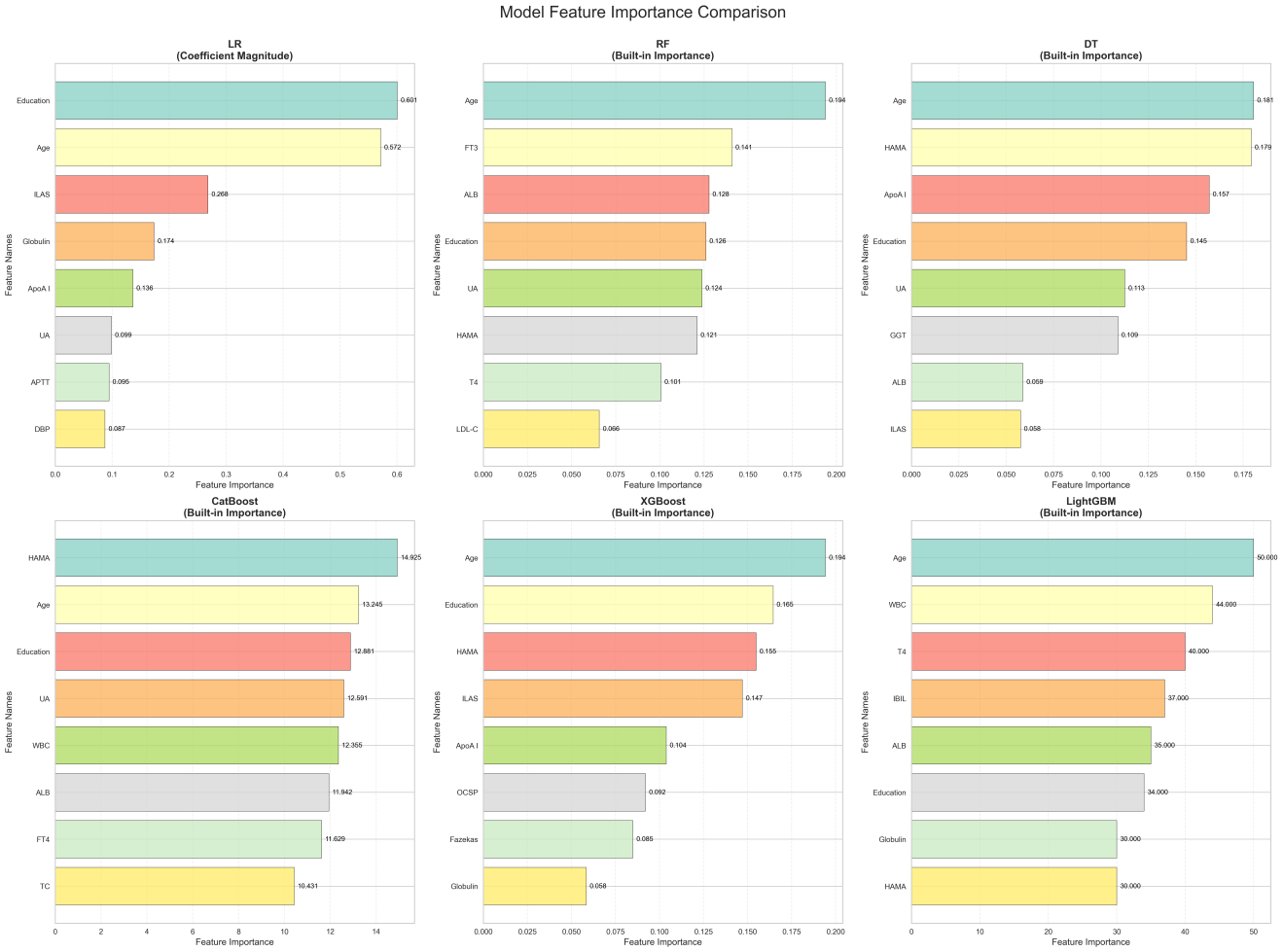


The figure displays the relative contribution of clinical variables to model performance across six algorithms, including LR, DT, RF, XGBoost, LightGBM, and CatBoost. Feature importance was determined by coefficient magnitude in LR and by built-in importance measures in tree-based models.

**Supplementary Figure 3. Feature Selection Correlation Heatmap**


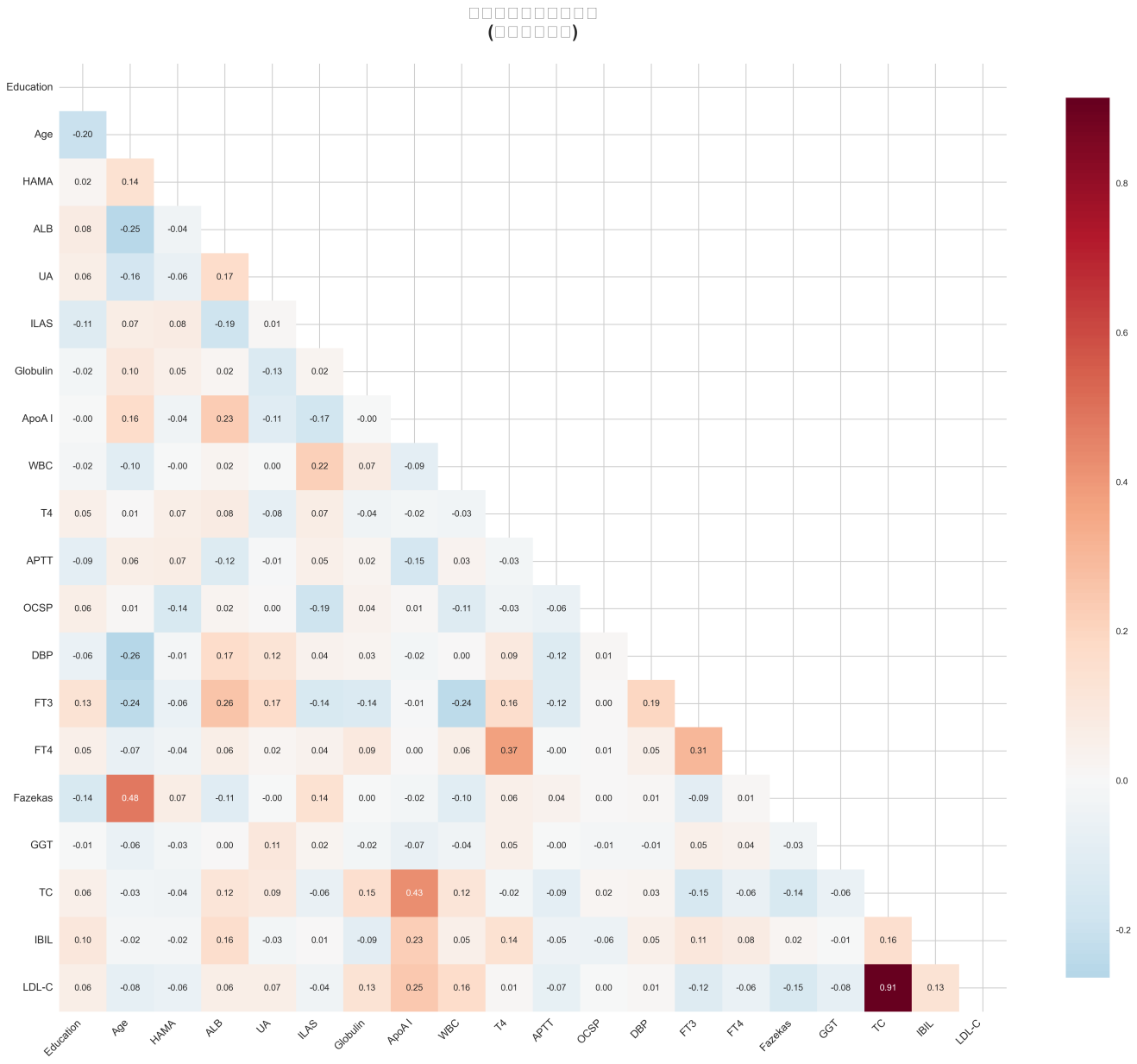


The heatmap illustrates pairwise Pearson correlation coefficients among all included demographic and biochemical variables. Warm colors indicate positive correlations, while cool colors indicate negative correlations. The strength of the correlation is reflected by color intensity, as shown in the accompanying color bar. Most variables exhibited weak-to-moderate correlations (|r| < 0.5), suggesting limited multicollinearity across predictors. Notably, TC and LDL-C showed a strong positive correlation, whereas thyroid-related indices (FT3, FT4, T4) demonstrated inter-variable dependency consistent with physiological coupling.
